# Word-Blindness

**Published:** 1888-01

**Authors:** 


### WORD-BLINDNESS.

In his "Treatise on Practical Medicine," published in 1838, Gendrin speaks of patients "who find it impossible to read, but who can write by a sort of memory of the movements of the fingers necessary to trace the words; when once the letter is written, the patient is no longer able to recognize it." An almost identical description is given by Trousseau, in a clinical lecture, of a form of aphasia first described and named as a distinct species by Kussmaul in 1877, and called by him *word-blindness* (Wortblindheit).

Charcot, in his last volume on "Diseases of the Nervous System," sums up the details of sixteen observations, of which one had been carefully studied by himself; these cases have all appeared in the medical journals the past few years. The following, in substance, are Charcot's conclusions: In general, the onset of this form of aphasia has been sudden, and attended, at first, with a certain degree of right hemiplegia, which soon disappeared. During the first few days there is almost always a little motor aphasia, which vanishes later, the word-blindness alone remaining. There are, however, exceptions to this, and, in a remarkable case, originally reported by Gueneau de Mussy, in Galezowski's *Journal Ophthamologique*, the word-blindness made its appearance isolated from all other complications of a paralytic kind. It is true, however, that a right-sided hemiplegia soon followed.

Visual troubles have been somewhat vaguely mentioned in connection with a certain number of cases. The patient whose case was reported by Westphal (*Zeitschrift für Ethnologie*, 1874), and the patient who was the subject of Charcot's description, had well-marked hemianopsia. So, also, with Gueneau de Mussy's patient.

A peculiarity of typical cases is that when the subjects of word-blindness make efforts to read a book or manuscript, they are obliged to trace out the letters and words with their fingers before they can obtain any understanding of what they are endeavoring to read. The ideas imparted by these movements of the fingers appear to be indispensable to give value and precision to the vague motions furnished by the visual images; in other words, the patient can only read by writing. In Charcot's patient, these digital movements, which were so necessary to supplement the sight in obtaining the recognition of words, were instinctive; in no other way, in fact, could the visual memories be recovered.

Charcot gives the result of three autopsies—one pertaining to a patient of M. Déjerine, another to a patient of M. Chauffard, the third to a patient of MM. Heilly and Chantêmesse. These three observations perfectly agree in one point—

the predominant lesion was found to be in the inferior parietal lobule, with or without participation of the angular gyrus and the first temporal convolution. In cases in which there was lesion of the latter convolution, the verbal blindness was found complicated with word-deafness. Charcot finds reason to conclude that the lesion on which verbal blindness depends has for its seat the inferior parietal lobule, with or without participation of the angular gyrus. The existence of the lesion in those parts of the cortex which cerebral physiologists have, with very general unanimity, fixed upon as the visual perceptive center, easily explains the hemianopsia which characterized several of the cases. As for the precise alteration by which verbal blindness is determined, we are still dependent on conjecture, although there is reason to believe that the primary vascular lesion is generally a plugging (thrombosis or embolism) of the Sylvian artery, which furnishes branches to Broca's convolution, the seat of motor aphasia, and to the regions where verbal blindness and hemianopsia seem to have their location. The lesion of these arterial branches is the primary fact, and the atrophy or softening of the cerebral tissue the consequence.

Gueneau de Mussy, who had an opportunity of studying a remarkable case, which is treated in his usual thorough and philosophical manner in the last volume which he published, proposed to substitute for the name *word-blindness* (*cécité verbale*), and the appellation *aphasic amblyopia*, which Galezowski had bestowed on this affection, the term *asémiognosie optique* (optical asémiognosia); that is, absence of the knowledge of signs. This term seemed to De Mussy more exact than that of *word-blindness*, for there is really no *blindness* present in the ordinary acceptation of the word, and the patient has lost the comprehension not only of words, but of letters. "In adding to the word *asémiognosia* the adjectives of visual or optic, acoustic or tactile, we may," says this writer, "express the different varieties of this intellectual trouble, or their combination." The term is a good one, though too cumbersome, and is not likely to supplant that originally proposed by Kussmaul.

To conclude, all studies thus far made in this department of cerebral physiology testify in favor of the division of the memory into several forms, which correspond to the impressions furnished by the different senses; "and it seems very probable that each of these forms, is in relation with a distinct department of the cerebral substance which is the material condition of the formation of these sensorial memories, of their conservation, and of their transmission without."—*Boston Medical and Surgical Journal.*

